# Hypothalamic Astrocyte Development and Physiology for Neuroprogesterone Induction of the Luteinizing Hormone Surge

**DOI:** 10.3389/fendo.2020.00420

**Published:** 2020-06-26

**Authors:** Kevin Sinchak, Margaret A. Mohr, Paul E Micevych

**Affiliations:** ^1^Department of Biological Sciences, California State University, Long Beach, Long Beach, CA, United States; ^2^The Laboratory of Neuroendocrinology, Department of Neurobiology, David Geffen School of Medicine at UCLA, Brain Research Institute, University of California, Los Angeles, Los Angeles, CA, United States

**Keywords:** neuroprogesterone, astrocyte, Src kinase, kisspeptin, RP3V, AVPV, estradiol, LH surge

## Abstract

Neural circuits in female rats sequentially exposed to estradiol and progesterone underlie so-called estrogen positive feedback that induce the surge release of pituitary luteinizing hormone (LH) leading to ovulation and luteinization of the corpus hemorrhagicum. It is now well-established that gonadotropin releasing hormone (GnRH) neurons express neither the reproductively critical estrogen receptor-α (ERα) nor classical progesterone receptor (PGR). Estradiol from developing ovarian follicles acts on ERα-expressing kisspeptin neurons in the rostral periventricular region of the third ventricle (RP3V) to induce PGR expression, and kisspeptin release. Circulating estradiol levels that induce positive feedback also induce neuroprogesterone (neuroP) synthesis in hypothalamic astrocytes. This local neuroP acts on kisspeptin neurons that express PGR to augment kisspeptin expression and release needed to stimulate GnRH release, triggering the LH surge. *In vitro* and *in vivo* studies demonstrate that neuroP signaling in kisspeptin neurons occurs through membrane PGR activation of Src family kinase (Src). This signaling cascade has been also implicated in PGR signaling in the arcuate nucleus of the hypothalamus, suggesting that Src may be a common mode of membrane PGR signaling. Sexual maturation requires that signaling between neuroP synthesizing astrocytes, kisspeptin and GnRH neurons be established. Prior to puberty, estradiol does not facilitate the synthesis of neuroP in hypothalamic astrocytes. During pubertal development, levels of membrane ERα increase in astrocytes coincident with an increase of PKA phosphorylation needed for neuroP synthesis. Currently, it is not clear whether these developmental changes occur in existing astrocytes or are due to a new population of astrocytes born during puberty. However, strong evidence suggests that it is the former. Blocking new cell addition during puberty attenuates the LH surge. Together these results demonstrate the importance of pubertal maturation involving hypothalamic astrocytes, estradiol-induced neuroP synthesis and membrane-initiated progesterone signaling for the CNS control of ovulation and reproduction.

## Introduction

Successful reproduction in female rodents depends on the interaction of steroidogenesis in the ovaries and brain. Almost 40 years ago Baulieu's group discovered that nervous tissue synthesizes steroids *de novo* from cholesterol and named them *neurosteroids* (1–4). Unraveling the physiology and actions of neurosteroids in the nervous system has been challenging because they are synthesized in specific locations, their actions must be differentiated from actions of circulating steroids, and in many cases the actions of peripheral steroids and neurosteroids are interdependent. Neurosteroids have been implicated in the myelination of peripheral nerves (5–8) neurogenesis (9) [reviewed in (10)], epilepsy, traumatic brain injury (11–13), and memory (14–18). Our research has concentrated on the role of the neurosteroid, neuroprogesterone (neuroP), which is synthesized *de novo* in hypothalamic astrocytes as part of the mechanism of estrogen positive feedback needed to stimulate the luteinizing hormone (LH) surge, inducing ovulation. This review considers estradiol signaling in the context of facilitating neuroP synthesis in astrocytes, and the integration of estradiol and neuroP signaling in regulating kisspeptin neurons in the rostral periventricular region of the third ventricle (RP3V). As with other steroid receptors, more recent findings indicate that in addition of nuclear localization and action, these receptors are trafficked to the plasma membrane where they are coupled to cell signaling cascades. The activation of nuclear progesterone receptor (PGR) at the cell membrane has recently been reviewed (19). In this review, we are primarily concerned with experimental evidence gathered in rodents. When appropriate, we indicate that the results were from different species. Kisspeptin is the most potent activator of neurons that release gonadotropin releasing hormone (GnRH) into the hypothalmo-hypophyseal portal circulation, generating a surge of pituitary LH into the systemic circulation. An LH surge is the trigger for ovulation and the formation of the corpus luteum—central events for reproduction.

## Positive Feedback, the LH Surge, and Ovulation

Hormones of the hypothalamic-pituitary-gonadal axis coordinate events that lead to maturation of ovarian follicles. The pivotal event is the LH surge that induces ovulation and reprograms the ovary to produce large amounts of progesterone as well as estradiol. These ovarian hormones are necessary to: (i) facilitate female sexual receptivity to maximize the potential of fertilization, (ii) induce the secretory phase of the stratum functionale completing the preparation of the uterine endometrium for implantation of the zygote should fertilization occur, and (iii) supporting the initial stage of pregnancy until the placenta develops.

Orchestrated actions of estradiol, progesterone and kisspeptin in the brain are critical for triggering the LH surge. GnRH neurons of the diagonal band of Broca (DBB) and medial septum project to the median eminence and release GnRH into the hypothalamo-hypophyseal portal system. GnRH regulates the release of follicle stimulating hormone (FSH) and LH from gonadotrophin cells in the anterior pituitary. Differential regulation of LH and FSH is accomplished by changes in GnRH release: low frequency and amplitude favor FSH release, whereas elevated amplitude and frequency preferentially release LH. Within the ovary, gonadotropins are critical for maturation of follicles, which become dependent on their stimulation. LH acts on the thecal and granular cells of the ovarian follicles and later the corpora lutea to regulate estradiol and progesterone synthesis throughout the cycle. At the beginning of the estrous cycle (diestrus I and II) as ovarian follicles mature, circulating estradiol levels slowly rise and produce negative feedback in the hypothalamus and pituitary retarding the release of gonadotropins. The main effects of negative feedback regulating GnRH release appear to be mediated through kisspeptin, neurokinin B, and dynorphin expressing (KNDy) neurons of the arcuate nucleus of the hypothalamus (ARH) (20) [reviewed in (21, 22)]. The mechanism of estrogen positive feedback requires the action of estradiol and progesterone, and yet, GnRH neurons do not express ERα or classical PGR (23–25). Therefore, estradiol and progesterone must signal through neurons upstream of the GnRH neuron. The majority of anterior hypothalamic kisspeptin neurons express ERα and PGR, providing a platform for integrating steroid actions that modulate the excitation of GnRH neurons (26–28). In rodents, positive feedback actions of steroids are mediated by kisspeptin neurons in the RP3V, which contains the anteroventral periventricular (AVPV) and rostral periventricular zone (25, 29–33). The AVPV is a site critical for estrogen positive feedback signaling in rodents. Lesioning or implanting anti-estrogens into the AVPV blocks the LH surge (34–36).

GnRH neurons in the DBB receive input from RP3V (including AVPV) kisspeptin neurons and are activated by kisspeptin to increase the frequency and amplitude of GnRH release inducing an LH surge from the pituitary (23–27, 29–31, 37–39). Infusion of exogenous kisspeptin excites GnRH neurons and induces levels of LH that mimic surge levels. GnRH neurons in the DBB express Kiss1R (formerly GPR54), the cognate receptor for kisspeptin (40–42). GnRH neuronal activation and the LH surge are lacking in female Kiss1R knockout mice (37). Activation of Kiss1R in GnRH neurons produces robust depolarizing currents and induces GnRH release (43–47). The timing of the LH surge requires the stimulatory action of kisspeptin and the removal of RFamide-related peptide 3 (RFRP-3; also known as gonadotropin-inhibitory hormone—GnIH) (48–53). In this model, the daily afternoon increase in GnRH and LH is due to suppression of the RFRP-3 inhibitory input to the GnRH neurons by the suprachiasmatic nucleus (SCN). We propose that estrogen positive feedback surge release of LH requires an amalgamation of circadian and kisspeptin models. It is only when the diurnal release of RFRP-3 inhibition of GnRH coincides with estradiol and neuroP stimulation of kisspeptin release that a GnRH–LH surge occurs–once every 4 days during the estrous cycle (52).

As estradiol levels rise rapidly and peak on the afternoon of proestrus, positive feedback predominates (54). Because estradiol treatments induce the LH surge in ovariectomized and adrenalectomized (OVX/ADX) rats, progesterone was not thought to be required for the LH surge and the phenomenon was called “estrogen positive feedback” (55, 56). A large number of studies unequivocally demonstrated that in addition to estradiol, “estrogen” positive feedback requires PGR and progesterone (55, 57–64). It turned out that the needed progesterone, neuroP, is synthesized in the hypothalamus (32, 65–68). Rising estradiol levels during diestrus 1 to proestrus induce the expression of PGR and kisspeptin in RP3V neurons that are critical for the LH surge (26, 28, 33, 37, 63, 69–71). This initial kisspeptin induction is dependent on ERα (70, 71). *In vivo* experiments did not differentiate between effects of estradiol that induced PGR and kisspeptin since both require ERα. Moreover, *in vivo* experiments did not segregate estradiol effects directly on kisspeptin neurons from neuroP-PGR effects on kisspeptin neurons. Our *in vitro* experiments allowed us to tease apart these overlapping effects. Proestrous (positive feedback) levels of estradiol stimulate hypothalamic astrocytes to synthesize neuroP that acts on the estradiol-induced PGR in kisspeptin neurons, which augments the synthesis and release of kisspeptin needed for the GnRH-LH surge (19, 32, 33, 65, 68, 72) [reviewed in (73)]. Thus, a critical component of positive feedback is estrogen-facilitated neuroP signaling through ERα and PGR expressing kisspeptin neurons.

## Synthesis of neuroP by Hypothalamic Astrocytes

**Figure 1:**
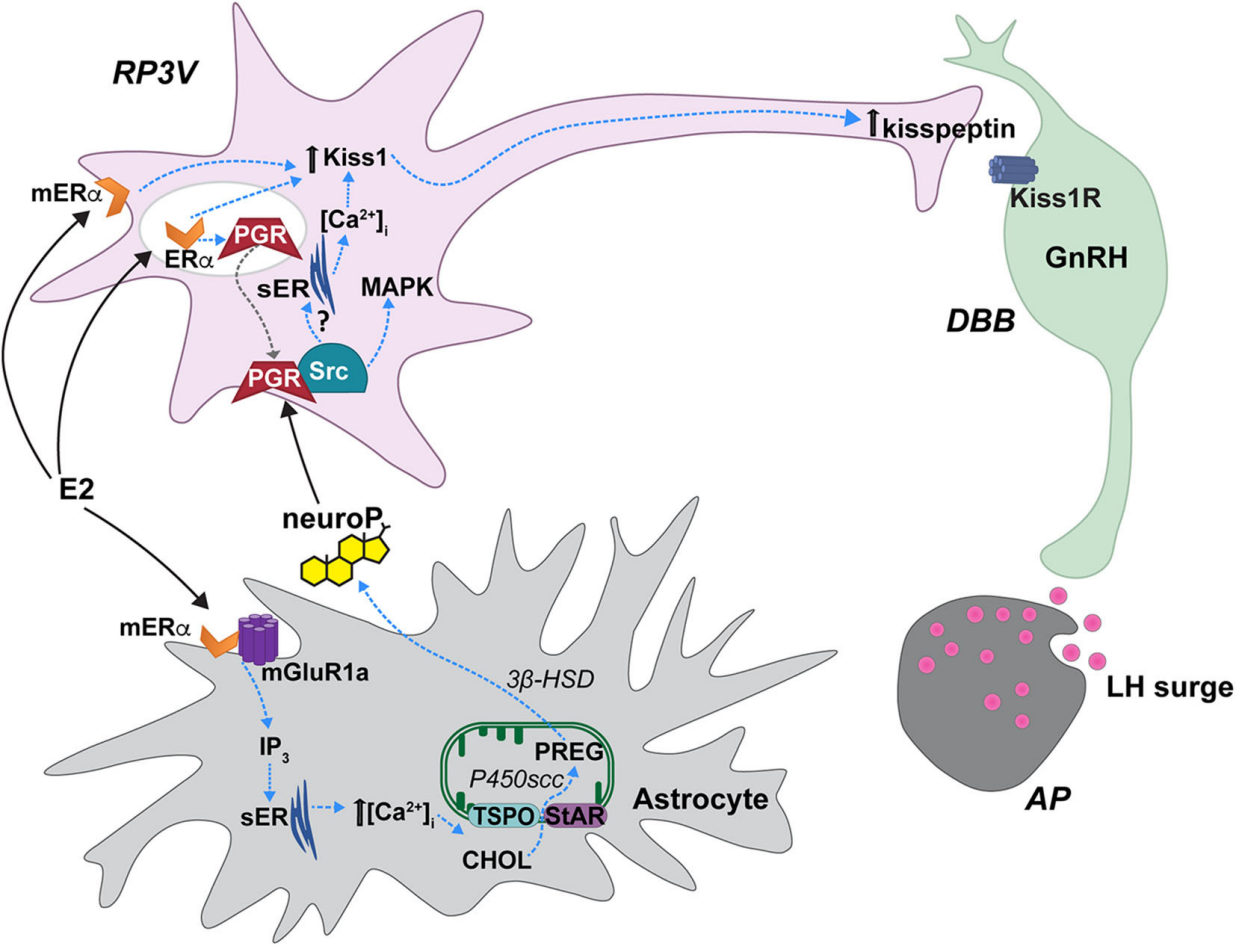
Proposed estradiol-induced hypothalamic astrocyte steroidogenesis of neuroP that activates a membrane classical progesterone receptor (PGR)-Src tyrosine kinase (Src) signaling pathway in RP3V kisspeptin neurons to trigger the luteinizing (LH) surge. In hypothalamic astrocytes, proestrous levels of estradiol (E2) activate membrane estrogen receptor-α (mERα) that complex with and signal through metabotropic glutamate receptor-1a type (mGluR1a). mERα-mGluR1a signals through a PKC-IP3 pathway to increase intracellular calcium concentrations [(Ca^2+^)]. This releases Ca^2+^ from the smooth endoplasmic reticulum (sER). Within the mitochondrion, cholesterol (CHOL) is converted to pregnenolone (PREG) by P450 side chain cleavage (P450scc). PREG is then converted to neuroprogesterone (neuroP) by 3β-hydroxysteroid dehydrogenase (3β-HSD). The neuroP is secreted from the astrocytes to activate ERα-mediated, E2-induced PGR in RP3V kisspeptin neurons. Concurrently, E2 increases Kiss1 mRNA and kisspeptin expression via a mERα initiated mechanism [but see (70)]. neuroP rapidly augments the E2-induced Kiss1 mRNA and kisspeptin expression, potentially through PGR-Src signaling. PGR complexes with and signals through Src to activate a MAPK pathway. Further, a membrane PGR can initiate signaling that increases intracellular Ca^2+^ from sER stores. PGR-Src signaling also mediates the release of kisspeptin from neurons that project to diagonal band of Broca (DBB) GnRH neurons. Kisspeptin then binds to its cognate receptor, Kiss1R stimulating GnRH release into the median eminence that triggers the LH surge from gonadotrophs in the anterior pituitary (AP). Steroid acute regulatory protein (StAR), translocator protein (TSPO). Modified from Micevych et al. (75).

Depending on the final bioactive steroid, neurosteroidogenesis may involve one or a combination of astrocytes, oligodendrocytes, and neurons (74). This is because each cell type expresses certain enzymes within the steroidogenic pathway (74). The *de novo* synthesis of neurosteroids that are further down the pathway from cholesterol (e.g., estradiol) require shuttling through multiple cell types in order to be synthesized. However, neuroP only requires two enzymes to be synthesized from cholesterol, and astrocytes express both of these enzymes (4, 74) (Figure 1). The synthesis of progesterone is initiated by transport of cholesterol into the inner mitochondrial membrane through the interaction of translocator protein (TSPO) and steroid acute regulatory protein (StAR) (76–78) [but see (79–81)]. Cholesterol is converted to pregnenolone by the enzyme CYP11A1 (previously P450 side chain cleavage; P450scc) that is associated with the inner mitochondrial membrane. 3β-hydroxysteroid dehydrogenase (3β-HSD or HSD3B1) converts pregnenolone to progesterone, which diffuses out of astrocytes to activate local PGR-expressing kisspeptin neurons of the RP3V (19, 82) facilitating the LH surge.

Positive feedback levels of estradiol induce neuroP synthesis in hypothalamic astrocytes. Proestrous levels of estradiol activate membrane ERα (mERα) that is complexed with and transactivates metabotropic glutamate receptor-1a (mGluR1a; Figure 1) to rapidly induce phosphorylation events that regulate cholesterol transport (65, 76, 83, 84). Estradiol activation of the mERα-mGluR1a complex, signaling through Gαq, activates the phospholipase C-inositol trisphosphate (IP3) signaling pathway that produces a robust release/increase of intracellular free calcium ([Ca^2+^]_i_) from intracellular stores (83, 85). This activates a calcium-sensitive adenylate cyclase-protein kinase A pathway that increases the phosphorylation of TSPO and StAR in hypothalamic astrocytes, which is necessary for inducing neuroP synthesis (83, 84, 86). These results suggest that proestrous levels of estradiol increase neuroP synthesis by increasing the cholesterol transport into mitochondria and access to P450scc for conversion to pregnenolone. *In vivo*, estradiol increases hypothalamic expression and activity of the second enzyme in the neuroP synthesis, 3β-HSD (85, 87). The estradiol-induced increase in brain progesterone levels are sex- and site-specific: present in the adult female hypothalamus but absent in the male hypothalamus (72). Moreover, blocking 3β-HSD activity in the hypothalamus of adult OVX/ADX rats prevents the estradiol-induced LH surge (72). We further demonstrated that neuroP is important for the LH surge in gonadally intact rats by blocking hypothalamic neuroP synthesis on the morning of proestrus by third ventricular (3V) administration of aminoglutethimide (AGT), a P450scc inhibitor (67). The estrous cycle is arrested in proestrus prior to the LH surge even though peripheral estradiol levels, a marker of ovarian steroidogenesis, are unaffected in AGT-treated rats. In these animals, the uterus is swollen with fluid and there are no corpora lutea in the ovaries—all indicating the absence of an LH surge (67). Thus, estradiol-induced hypothalamic neuroP, rather than ovarian or adrenal progesterone, mediates the triggering of the LH surge during positive feedback. Dose and duration of estradiol exposure during negative and positive feedback regulate the mechanisms of neuroP synthesis by astrocytes, and properly coordinate the timing of neuroP synthesis with the priming of the rest of the Kisspeptin-GnRH-LH system.

## neuroP Signaling Through Membrane PGR to Regulate Kisspeptin

neuroP actions appear to be mediated through PGR signaling in RP3V kisspeptin neurons. Estradiol-induced RP3V PGRs are required to initiate and reach the full magnitude and duration of the LH surge (62, 88). Likewise, the LH surge cannot be induced in PGR knockout mice (89). Female mice with PGR knocked out specifically in kisspeptin neurons are less fertile (i.e., fewer births with smaller litters), and lack an estradiol-induced LH surge and the associated AVPV c-Fos induction (90, 91). Furthermore, activation of RP3V PGR with R5020 (PGR specific agonist) induced an LH surge in estradiol-primed rats (33). Accumulating evidence supports that it is neuroP signaling through PGR in kisspeptin neurons that is required for the LH surge. While blocking neuroP synthesis with a 3β-HSD inhibitor attenuates the estradiol-induced LH surge in OVX/ADX rats, progesterone treatment or site-specific injections of kisspeptin into the DBB rescued the LH surge, demonstrating that estradiol induction of neuroP synthesis and the actions of neuroP occur first and are required for kisspeptin release (32).

**Figure 2:**
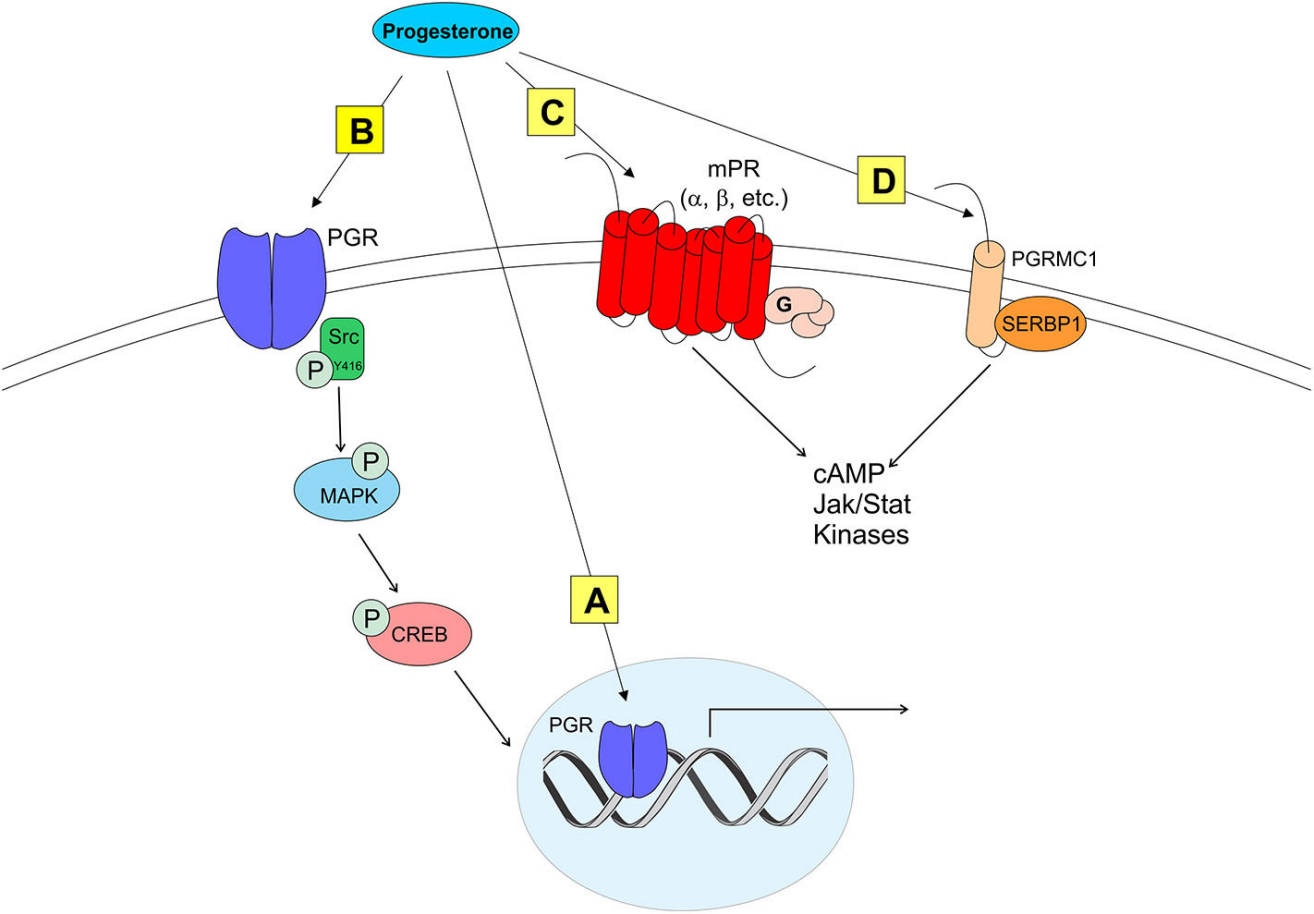
Modes of progesterone signaling in the rodent. Classical progesterone receptor (PGR) can mediate progesterone signaling classically **(A)**, by binding to DNA progesterone response elements. PGR can also be trafficked to the plasma membrane [as in **(B)**] where it can activate rapid intracellular signaling cascades involving kinases such as Src. It is unknown whether membrane PGR transactivates another receptor like an mGluR as estrogen receptors have been shown to do. Multiple novel membrane progesterone receptors (mPRs) have been recently discovered and described, such as mPRs α, β, δ, and γ **(C)**. mPRs can activate signaling cascades via G proteins, which go on to affect cyclic AMP (cAMP) pathways. Finally, progestins can bind to progesterone receptor membrane component 1 [PGRMC1 **(D)**]. PGRMC1 can work in concert with SERBP1 to affect cAMP, Jak/Stat, and multiple kinase pathways (73).

RP3V kisspeptin neurons are modeled *in vitro* using mHypoA51 cells that are derived from adult female hypothalamic kisspeptin neurons (19, 82). Estradiol induces PGR expression in these cells (19), which is observed in the RP3V where PGR expression is increased in areas that overlap with kisspeptin neurons by estradiol treatment in OVX rats or on proestrus (28, 33). Estradiol increases kisspeptin expression in mHypoA51 neurons, and subsequent progesterone further augments this expression (19). In co-culture experiments where mHypoA51 and adult female hypothalamic astrocytes are separated (i.e., not in direct contact) but share media, estradiol treatment induces neuroP synthesis in astrocytes and increases kisspeptin expression in the mHypoA51 neurons. neuroP secretion from astrocytes stimulates mHypoA51 neurons to rapidly increase kisspeptin release (19, 82). Importantly, mHypoA51 neurons express membrane progesterone receptors (mPR), including mPRα, mPRβ (see more below and Figure 2), and membrane-localized PGR. This membrane-localized PGR increases with estradiol treatment (19).

Although classified as a transcription factor and normally thought to be associated with the nucleus, PGR can be trafficked to the plasma membrane via palmitoylation, a mechanism seen in ERα trafficking (92). At the membrane, PGR can interact with and signal through other proteins to initiate rapid signaling, altering neuronal activity (33, 93, 94) (Figure 2). PGRs that are trafficked to the plasma membrane complex with and signal through Src kinase, a non-receptor tyrosine kinase (Src) (33, 93–95). PGRs have two distinct isoforms that are transcribed from a single gene: PGR-A and PGR-B. PGR-A lacks 164 amino acids in the N-domain, and is considered the truncated form of PGR-B (96). A poly-proline rich region (amino acids 421-428, PPPPLPPR) near the N-domain of PGRs is responsible for binding and signaling through the Src SH3 domain (93). Although this region is conserved in PGR-A, and both isoforms display hormone-dependent binding to SH3, only PGR-B activates Src (94). Significantly, PGR-B is also the reproductively relevant isoform.

*In vitro* and *in vivo* experiments indicate that neuroP signals through PGR-Src complexes to activate kisspeptin neurons, and that the PGR-Src signaling is interdependent. Nearly all mHypoA51 neurons express kisspeptin, and most express PGR and Src (19, 82). Activation of either PGR or Src in mHypoA51 neurons induces kisspeptin release while inhibiting Src activation blocks progesterone activation of MAPK and kisspeptin release, implying that progesterone and Src interact to stimulate kisspeptin release via activation of a MAPK pathway (Figure 1) (19). Another potential rapid PGR initiated pathway for kisspeptin release is through release on intracellular stores of [Ca^2+^] (Figure 1) (82). In mHypoA51 neurons, progesterone induced a rapid increase in [Ca^2+^] that was blocked by pretreatment with RU486, a PGR antagonist (82). However, further studies are required to determine the physiological outcomes of both of these signaling pathways. *In vivo* data further support that neuroP induces Src-mediated PGR signaling. PGR and Src are co-expressed in neurons of the RP3V of female rats (33). Further, using the Duolink proximity ligation assay that uses specific antibodies to two selected proteins/antigens and then produces punctate staining if these proteins are in close proximity (<40 nm), we observed that estradiol-priming increases the levels of PGR and Src staining in RP3V neurons suggestive of an estradiol-induced increase in PGR-Src interactions (33). Similarly, in the ARH, a region important for facilitation of lordosis, we have observed a similar colocalization and estradiol-induced increase in PGR-Src proximity (97). In the RP3V, PGR and Src exhibit interdependent signaling in the induction of the LH surge. Bilateral infusion of either a classical PGR agonist (R5020) or Src family activator induced a robust LH surge in estradiol-primed OVX/ADX rats (97). However, bilateral RP3V infusion of either a PGR antagonist (RU486) or a Src inhibitor (PP2) blocked the induction of the LH surge by activation of either PGR (progesterone or R5020) or Src (Src activator). The ability of antagonizing either PGR or Src to block the signaling of both PGR and Src indicates that PGR-Src signaling is interdependent. It is likely that PGR is transactivating Src to initiate signaling. Even though Src is “downstream” of PGR, and activation of either one will induce the LH surge, for signaling to occur neither can be occupied by an antagonist, which likely produces a conformational change that prevents Src activation and signaling. This antagonist effect was also seen with interactions of PGR and dopamine receptors (98). However, in the absence of antagonist binding either PGR or Src, it appears the activation of either PGR or Src can initiate the signaling cascade. The similarities of the PGR-Src signaling cascade in the ARH, RP3V and mHypoA51 neurons suggests that PGR-Src signaling may be a common mode of membrane PGR signaling. Together, the *in vivo* and *in vitro* findings indicate that membrane PGR-Src signaling mediates the neuro P activation of kisspeptin neurons to activate GnRH neurons to trigger the LH surge.

## Non-Classical Progesterone Receptors in Kisspeptin Neurons

Although PGRs are essential to induce the LH surge, other types of mPRs have been proposed to modulate neuroP/progesterone actions through membrane initiated signaling [reviewed in (73); see Figure 2]. For example, during progesterone negative feedback, PGR knockout mice respond to positive feedback levels of progesterone to suppress GnRH release, suggesting that progesterone may also signal through non-classical mPRs (99). However, the role of non-classical mPR in the LH surge remains unknown (19, 100). Two families of mPRs have been discovered that initiate progesterone signaling at the plasma membrane. One group of these mPRs is in the Class II progestin and adipoQ receptor (PAQR) family (101–103). These mPRs have a classic 7-transmembrane protein structure and behave similarly to G protein-coupled receptors by rapidly facilitating progesterone action. *In vitro*, mHypoA51a neurons express subtypes of mPRs: mPRα and mPRβ (19). *In vivo*, estradiol upregulates mPRβ expression in the anterior hypothalamus (104). The distribution and estrogen regulation of mPRβ in the female rat brain (104), but not in mHypoA51 neurons (19), suggests that this *in vivo* upregulation occurs in non-kisspeptin cells. Although these mPRs are expressed in the RP3V, little is known about the role of these non-classical mPRs in regulating the neuroP induction of the LH surge. Another protein that binds progesterone and initiates signaling at the plasma membrane is progesterone receptor membrane components (PGRMC) (105–109). Two PGRMC subtypes have been discovered: PGRMC1 (aka 25-DX) and PGRMC2 (105, 106, 108, 110, 111). These PGRMC have been implicated in normal mammalian ovarian function including primordial follicle development, luteal vascularization and normal onset reproductive senescence (107, 110, 112). Young women with reduced PGRMC2 expression in granulosa cells have been diagnosed with diminished ovarian reserve (113). Similarly, reduced expression of PGRMC1 (via point mutation) has been associated with women exhibiting primary ovarian insufficiency (114). PGRMC mRNAs are expressed in the AVPV (109, 111, 115). However, only PGRMC2 mRNA levels are upregulated by the sequential treatment with estradiol and progesterone (115). Although their expression in the AVPV does not appear to be essential, mPRs and PGRMC's may influence PGR actions. A resolution of this issue requires further experimentation.

## Development of Estrogen Positive Feedback During Puberty

Maturation of reproductive circuits in females that results in ovulation involves a multitude of changes in the brain during puberty. This is represented by the increase in GnRH pulsatility throughout puberty, which is needed for the surge release of LH [reviewed in (116)]. As with estrogen positive feedback and the facilitation of neuroP synthesis, kisspeptin neurons in the rodent AVPV are sexually dimorphic; females have more kisspeptin neurons in the AVPV compared with males (30). In female mice, the number of presumptive kisspeptin neurons in the AVPV increases across pubertal development (117). The increase in GnRH pulsatility and the increase in the number of kisspeptin neurons are crucial to the development of estrogen positive feedback, but the maturation of these two systems do not fully explain the development of estrogen positive feedback during puberty.

## Pubertal Development of Estradiol-Induced neuroP Synthesis in the Hypothalamus

Much like the rest of the system that controls estrogen positive feedback, the ability of the hypothalamus to coordinate estradiol-induced neuroP synthesis is something that develops across puberty in the rodent. It was previously observed that primary hypothalamic astrocyte cultures did not increase progesterone synthesis in response to estradiol if harvested from neonatal female or male mice of any age, and maturation *in vitro* did not make these astrocytes respond to estradiol with neuroP synthesis (65). At the time it was thought that something about the pubertal transition made astrocytes competent to respond to estradiol, but this idea was not formally tested until recently. Mohr et al., showed that estradiol-facilitated neuroP synthesis in the hypothalamus develops during puberty in the female rat (118). First, hypothalamic tissues, collected from gonadally intact rats either on postnatal day 17, (PND 17; prepuberty), PND 35, (peripuberty), or on the afternoon of proestrus around PND 60 (adulthood), were assayed for neuroP levels with liquid chromatography tandem mass spectrometry (LC-MS/MS). NeuroP significantly increases during puberty in gonad-intact female rats, from prepuberty to adulthood. Then, in OVX rats of the same ages, estradiol treatment only in adulthood reliably facilitates neuroP synthesis. The prepubertal female hypothalamus is insensitive to estradiol in terms of neuroP synthesis. However, during puberty (peripuberty) the ability of estradiol to stimulate neuroP synthesis develops. The pubertal development of neuroP synthesis is yet another way that the brain changes during puberty to allow for estrogen positive feedback signaling.

Adult female hypothalamic astrocytes are the source of estradiol-facilitated neuroP synthesis that contributes to estrogen positive feedback (68). Corresponding to the pubertal increase in estradiol-facilitated neuroP synthesis *in vivo*, there is an increase in the amount of mERα in hypothalamic astrocyte cultures. In these primary female astrocyte cultures, there is also an increase in caveolin-1 protein, a scaffolding protein that participates in the trafficking of ERα to the cell membrane and coupling with mGluR1a (118). Because membrane-initiated estradiol signaling is necessary to augment neuroP synthesis in astrocytes in adulthood [reviewed in (119)], the lack of mERα provides an explanation as to why pre-pubertal hypothalamic astrocytes are incapable of estradiol-induced neuroP synthesis. It appears that pubertal expression of caveolin-1 that shepherds ERα to the membrane may be key to the development of estrogen positive feedback that induces neuroP synthesis to trigger the LH surge.

## Pubertally Born Astrocytes in the AVPV: Key to the Development of neuroP Synthesis?

**Figure 3:**
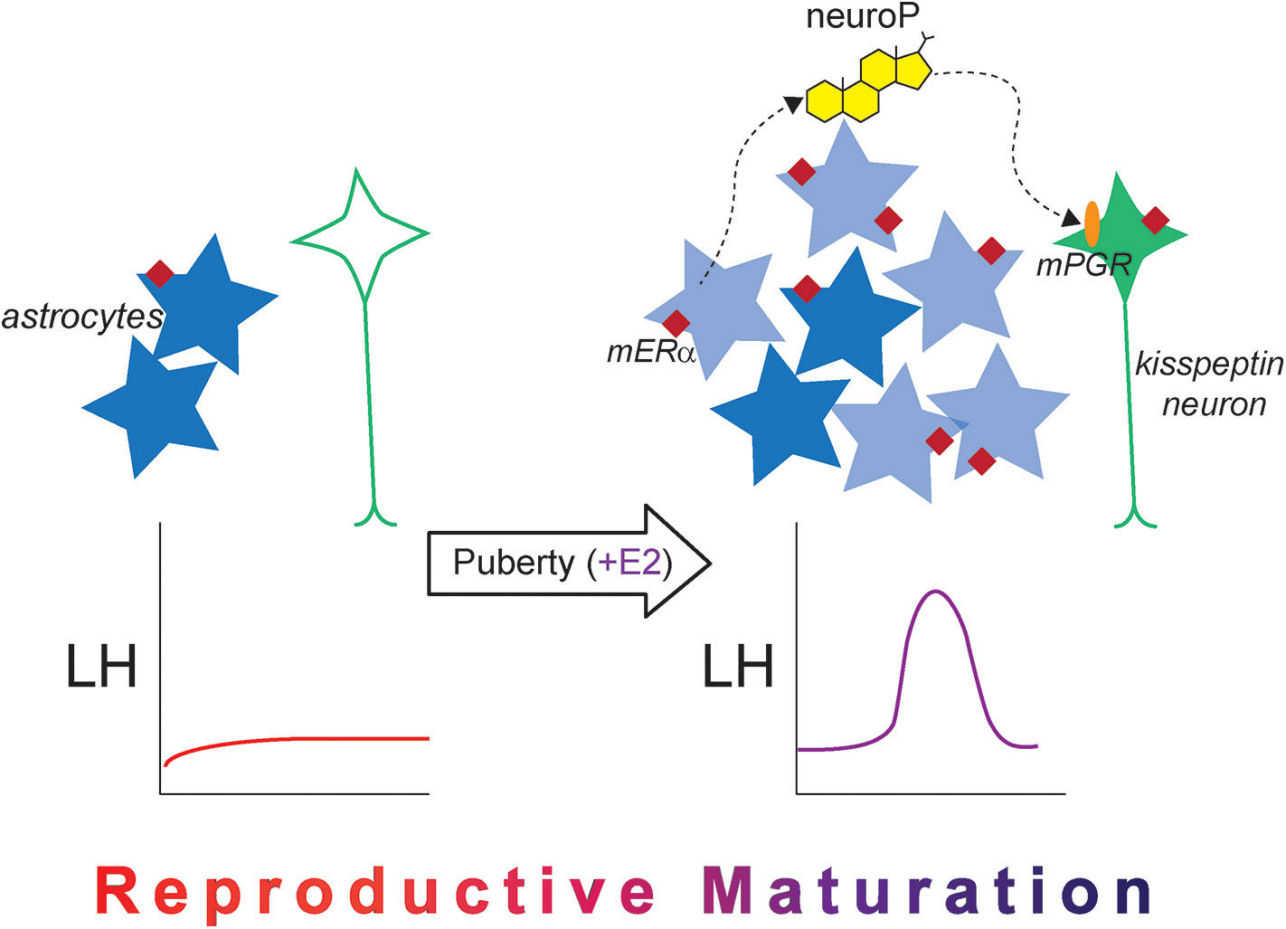
The new astrocyte model for the pubertal development of rodent estrogen positive feedback. Prior to puberty, kisspeptin expression is low in the female AVPV (green neuron), and mERα expression (maroon diamond) in hypothalamic astrocytes (dark blue stars) is low. The LH surge does not occur naturally and cannot be induced with exogenous estradiol administration. After puberty occurs and once estrogen signaling begins, estrogen-responsive newborn astrocytes are added to the AVPV (light blue stars), which are necessary for the LH surge in adulthood. There is also an increase in mERα expression in hypothalamic astrocytes, and estradiol-induced neuroP synthesis occurs, which acts on membrane-associated PGR (orange oval) to stimulate kisspeptin release. Together, the changes in cellular machinery across pubertal development alter estradiol responsiveness and permit the LH surge to occur, indicating that reproductive maturation is complete.

Another explanation of how estradiol-induced neuroP synthesis develops in the hypothalamus is the “new astrocyte model” (Figure 3). Accordingly, estradiol-responsive astrocytes are not present in the prepubertal hypothalamus. During and after puberty, populations of new cells are added to the female rat AVPV, and a large majority of these newly born cells express markers for astrocytes (GFAP) (120, 121). These newborn AVPV cells are more numerous in females compared with males, and this sex difference in pubertal cell addition is dependent on gonadal hormones (122). Pubertal cell addition to the AVPV mirrors the overall sex difference observed in the rodent AVPV, considering that the female AVPV is larger and contains more neurons in females compared with males (123). This sex difference in structure likely contributes to the functional sex difference of this brain region, because only female rodents are capable of estrogen positive feedback (124). That females have higher amounts of cells added to the AVPV compared with males during peripuberty may indicate that these newborn cells are needed for estrogen positive feedback signaling.

Indeed, these newly born cells are crucially important for estrogen positive feedback induction of the LH surge. When cell proliferation is blocked with cytrabine (AraC), a pyrimidine analog, either during puberty or in early adulthood, the estradiol + progesterone-induced LH surge is diminished in female rats (120). A majority of these newborn cells are astrocytes, suggesting that these newborn AVPV astrocytes are the source of estradiol-responsive hypothalamic astrocytes that synthesize neuroP necessary for estrogen positive feedback. In the 2017 study by Mohr et al., both estradiol and progesterone were used to elicit the LH surge (120). In this study, the LH surge was not eliminated entirely, which may be explained by several factors: AraC did not eliminate *all* newborn AVPV cells, and therefore, some neuroP-producing astrocytes were present to produce neuroP and elicit some LH release, or more likely, administration of progesterone on the morning of the day of the surge bypassed hypothalamic neuroP, eliciting some LH release. Had only estrogen been used, which can also elicit the LH surge in rats (72), the effect of AraC may have been more dramatic on the LH surge because the only source of progesterone would have been from hypothalamic astrocytes (neuroP).

More studies are required to determine the exact role of pubertally born astrocytes in the development of estrogen positive feedback signaling. However, it seems likely that the birth of astrocytes contributes to the maturation of reproductive circuits controlling estrogen positive feedback signaling. There may be a developmental difference in the birth and maturation of astrocytes if they are born while circulating estradiol is elevated (i.e., after puberty compared with before puberty) that makes them competent to respond to estradiol with neuroP synthesis. These newborn astrocytes could have higher levels of mERα, and caveolin-1, resulting in increased PKA phosphorylation, making them more proficient in estradiol-induced neuroP synthesis.

## Conclusions

The regulation of ovulation is the central event in mammalian reproduction and during puberty. Indeed, neural circuits controlling reproduction in females are considered mature when ovulation can occur. In rodents, at the very least, this critical physiological process requires the coordination of the hypothalamo-pituitary-ovarian axis with the SCN circadian clock. Regulation of the surge release of LH requires a complex neuronal and glial circuitry that directs various peripheral and central hormones onto kisspeptin neurons. In turn, circadian-regulated inputs interact with GnRH neurons, activating the anterior pituitary to release a surge of LH. This mechanism has been dubbed “estrogen positive feedback” for the importance of estrogen, but it is far from the only critical hormonal participant in this process. Developing ovarian follicles synthesize ever increasing levels of estradiol that induce PGR expression in kisspeptin neurons. As estradiol levels peak on the afternoon of proestrus, neuroP synthesis is rapidly facilitated in hypothalamic astrocytes, many of which may be born after the initiation of puberty. Together, estradiol and neuroP stimulate kisspeptin expression and release. When this hormonal activation of kisspeptin coincides with the release of circadian inhibition–a physiological LH surge occurs (i.e., one that stimulates ovulation). We now understand the signaling involved in regulating both the synthesis of neuroP in astrocytes and the neuroP signaling in kisspeptin neurons. In astrocytes, mERα transactivates mGluR1a to induce neuroP synthesis. In kisspeptin neurons, a portion of estradiol-induced PGR are trafficked to the cell membrane where neuroP activates them, augmenting both kisspeptin expression and release. NeuroP signaling in kisspeptin neurons involves Src activation and the release of intracellular calcium. Thus, the brain does not passively respond to ovarian hormones but is an active participant in triggering the LH surge to induce ovulation.

## Author Contributions

All authors contributed to the writing and editing of this manuscript.

## Conflict of Interest

The authors declare that the research was conducted in the absence of any commercial or financial relationships that could be construed as a potential conflict of interest.
